# An UPLC-Q-TOF/MS-Based Analysis of the Differential Composition of Dendrobium officinale in Different Regions

**DOI:** 10.1155/2022/8026410

**Published:** 2022-11-04

**Authors:** Qianqian He, Anjing Lu, Lin Qin, Qianru Zhang, Yanliu Lu, Zhou Yang, Daopeng Tan, Yuqi He

**Affiliations:** ^1^Key Laboratory of Basic Pharmacology of Ministry of Education and Joint International Research Laboratory of Ethnomedicine of Ministry of Education, School of Pharmacy, Zunyi Medical University, Zunyi 563000, Guizhou, China; ^2^Shanghai Nature-Standard Technical Service Co.,Ltd, Shanghai 201203, China

## Abstract

*Dendrobium officinale* (*D. officinale*) is a valuable traditional Chinese herbal medicine with high commercial value. In *Chinese Pharmacopoeia* (Ch.P., 2020 edition), the quality of *D. officinale* is mainly evaluated by its polysaccharide content. However, varying growth and production conditions, such as cultivation environment, origin, harvesting process, or processing methods, resulting in highly variable yields, quality, and composition. The aim of this study was to investigate whether the content of secondary metabolites in *D. officinale* from different origins is consistent with the polysaccharide content. The results showed that the polysaccharide content and pass rate were ranked as GX > AH > GZ > YN. Based on the nontargeted metabolomics approach, we searched for differential components in 22 different regions of *D. officinale*, including amides, bibenzyls, disaccharide, flavonoids, organic nitrogenous compounds, and phenolic glycosides. The overall expression was opposite to the polysaccharide, and the most expressed was YN, followed by GZ, AH, and GX. These results indicated that the current quality standard for evaluating the quality of *D. officinale* by polysaccharide content alone is imperfect, and small molecule compounds need to be included as quality markers.

## 1. Introduction

The genus *Dendrobium* is one of the largest genera of the *Orchidaceae*, which contains 1500–2000 species [1]. Among them, *Dendrobium officinale* Kimura et Migo is the major source of Dendrobii caulis, a traditional Chinese medicine, which is widely distributed worldwide, such as the United States, Australia and Japan [2]. Especially, *D. officinale* is widely cultivated in various regions of China, including Anhui, Zhejiang, Hunan, Fujian, Guangxi, Sichuan, and Yunnan provinces [3–5].

*D. officinale* is a traditional Chinese medicine first recorded in the “Shen Nong's Herbal Classic” and used alone or as a prescription [6]. According to the practitioner of Chinese medicine's consensuses, the fresh or dried stems of *D. officinale* are considered to be a drug of nourishing Yin, which comprises the efficacy of nourishing the stomach for promoting the production of fluid, nourishing Yin and clearing heat, brightening the eyes, strengthening the waist and tonifying lung and kidney [7]. In addition, modern pharmacological studies have revealed that *D. officinale* has multiple promising bioactivities, including immunomodulation, antitumor, antioxidant, antifatigue, glycolipid regulation, hepatoprotection, etc [8–11]. According to current phytochemical investigations, more than 190 compounds have been isolated from *D. officinale*, including polysaccharides, alkaloids, amino acids, flavonoids, trace elements, and other nutrients, and its medicinal components are mainly composed of polysaccharides, bibenzyl, phenanthrene, flavonoids, and alkaloids [12–15]. Among them, polysaccharide was selected as the only quality marker in the current edition of the Ch.P., the relationship between polysaccharide and other small molecular components content is not yet known.

Due to the overexploitation and depletion of wild plant resources, D. *officinale* has become one of the rarest and most endangered Chinese herbal medicines in China, step by step [16,17]. In addition, with the current quality standards, the quality of *D. officinale* varies greatly from different region and growing environment [18]. Fortunately, the manual cultivation of *D. officinale* has made a great breakthrough, and currently *D. officinale* was mainly grown in greenhouses with the advantages of fast growth, high yield, and stable production, and became the most important source of the traditional Chinese medicine Dendrobii caulis. The components in Chinese herbal medicine are very complex. Thus, the quality of *D. officinale* cannot yet be accurately evaluated by its polysaccharides content alone. Therefore, the present study proposal explores the relationship between small molecular components and polysaccharides in *D. officinale* from greenhouse cultivation in different regions (AH, GX, GZ, and YN). To provide a theoretical basis for the collection, conservation, and utilization of germplasm resources of *D. officinale* in different regions.

## 2. Materials and Methods

### 2.1. Chemicals

LC-MS grade acetonitrile, methanol, formic acid, and water were purchased from Merck (Darmstadt, Germany), CNW Technologies GmbH (Duesseldorf, Germany), and Watsons (Hong Kong, China), respectively, for the LC-MS analyze. The reagents required for the polysaccharide assay including anhydrous ethanol, phenol, concentrated sulfuric acid, and glucose standards were purchased from Kelong Chemical Reagent Factory (Chengdu, China), Sinopharm Chemical Reagent Co., Ltd (Shanghai, China), and Merck (Darmstadt, Germany), respectively.

### 2.2. Sample Preparation

A total of 71 samples of *D. officinale* were collected from four regions, including *D. officinale* from Huoshan County of Anhui province (AH), *D. officinale* from Rong County of Guangxi province (GX), *D. officinale* from Danzhai County of Guizhou province (GZ) and *D. officinale* from Menglian County of Yunnan province (YN), in 2019 (see Table 1). The fresh stems of the 3-year-old samples were dried at 60°C, grounded into fine powder and stored at −80°C for the assay.

### 2.3. Determination of Polysaccharides

The quantification of polysaccharides in *D. officinale* follows the guidance of the Ch.P. (2020 edition) [6]. Firstly, a calibration curve was prepared by each UV absorbances(y) against concentrations (*x*, *μ*g/mL) of glucose standard. The linear regression equation was showed as y = ax + b. And then, the *D. officinale* powder sample (0.06 g) was heated and refluxed in a tested round bottom flask for 2 h. After cooling, it was precipitated with anhydrous ethanol for 1 h, then washed with 80% ethanol and the precipitate was dissolved with hot water to obtain the sample to be tested. Then, the phenol-sulfuric acid reaction was performed: 5% phenol solution (1.0 mL) and concentrated sulfuric acid (5.0 mL) were added sequentially to the sample to be tested (1.0 mL), rapidly shaken, heated in boiling water for 20 min, immediately removed and ice bathed for 5 min. The absorbance of the reaction solution was measured at 488 nm and the polysaccharide content was calculated according to the glucose standard calibration curve.

### 2.4. Metabolite Extraction

Dried and crushed *D. officinale* powder (75 mg) was weighed precisely, dispersed in 1 mL of 70% methanol, extracted by ultrasonication (400 W, 50 kHz) for 30 min, cooled, centrifuged at 12000 rpm for 5 min, and the supernatant was taken to analyze the secondary metabolites of *D. officinale* by UPLC-Q-TOF/MS.

### 2.5. UPLC-Q-TOF/MS Conditions

Samples were evaluated using a Waters 1290 Infinity II UPLC liquid chromatograph system (Waters Corp., Milford, MA, USA) equipped with a binary pump, in-line degasser, autosampler, and thermostatically controlled column chamber. The separation of an aliquot of 1 *μ*L sample solutions was performed on a Waters CORTECS UPLC C_18_ (100 mm × 2.1 mm, 1.6 *μ*m) maintained at 40°C. The mobile phase was solvent consisting of solvent A (0.1% formic acid in water, v/v) and solvent B (0.1% formic acid in acetonitrile, v/v) with a flow rate of 0.4 mL/min and a separation time of 10 min. Gradient elution conditions: 0–0.5 min, 5% B; 0.5–4 min, 40% B; 4–5 min, 75% B; 5–5.1 min, 95% B; 5.1–6.5 min, 95% B; 6.5–6.6 min, 5% B; 6.6–10 min, 5% B. The injection volume was 1 *μ*L.

Mass spectrometry was performed using an Agilent Q- TOF/MS system (Agilent, MA, USA), with separate acquisitions in positive and negative ion mode. Ion source parameters: mass scan ranged m/z 50 to 1200; gas temperature, 350°C; dry gas flow rate, 10 L/min; nebulizer, 45 psig; shealth gas temperature, 350°C; sheath gas flow rate, 11 L/min; vcap voltage, 4000 V; nozzle voltage, 1000 V; fragmentor, 175 V. Secondary mass spectrometry information was acquired using Auto MS/MS mode with CE of 20, 30, and 40 V, respectively. Data acquisition and processing were performed by Agilent Mass Hunter Profinder analysis software.

### 2.6. Data Processing and Analysis of Secondary Metabolites

Peak matching, peak alignment, ion fusion, and deconvolution were performed on the raw data using Agilent Mass Hunter Profinder software (version 10.0). Fragmented peaks with false positives were excluded based on peak area, retention time, and molecular weight. The analysis was performed by unsupervised pattern recognition principal component analysis (PCA) and projections on the latent structure-discrimination analysis (PLS-DA) models of SIMCA 14.1 software, with variable importance in the projection (VIP) value greater than 3 was used as a threshold to screen differential compositions. The ggplot2, pheatmap, and other packages were applied in the R program (version 4.1.1) for other visualizations. *p* < 0.05 was considered statistically significant.

## 3. Results

### 3.1. Comparison of Polysaccharide Content in *D. officinale* in Different Regions

**Figure 1:**
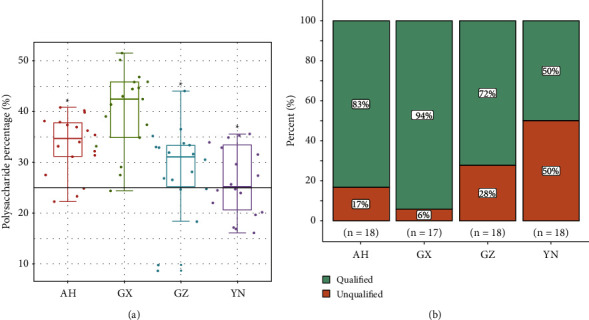
The polysaccharide content assessment of *D. officinale* from four regions. (a) Polysaccharide content of *D. officinale* from four regions. ^*∗*^ indicates significant difference compared to GX (*p* < 0.05). (b) Polysaccharide qualification rate of *D. officinale* from four regions.

The polysaccharide contents of 71 samples collected from four regions were determined. The Ch.P. stipulates that *D. officinale* polysaccharide content of not less than 25% is qualified. The results showed (see Figure 1) that the average polysaccharide contents in *D. officinale* of the four regions AH, GX, GZ, and YN were 33.51%, 40.11%, 28.39%, and 26.26%, and their passing rates were 83%, 94%, 72%, and 50%, respectively. The polysaccharide content of *D. officinale* in GX was significantly higher than that of other places.

### 3.2. Secondary Metabolomics of *D. officinale* in Different Regions

**Figure 2:**
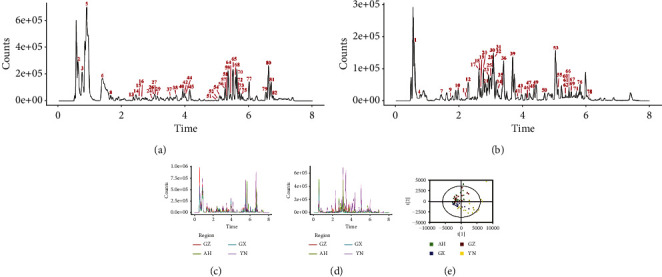
Profile analysis of secondary metabolomics of *D. officinale* in different regions. The quality control and typical base peak chromatogram of *D. officinale* in positive ion mode (a, c) and negative ion mode (b, d). PCA score plot of *D. officinale* from four regions (e).

The metabolic information of *D. officinale* from four regions was investigated by nontargeted metabolomics techniques. The base peak chromatogram (BPC) of quality control samples showed retention times mainly within one to eight min in positive ion (e.g., in Figure 2(a)) and negative ion (e.g., in Figure 2(b)) modes. A total of 82 metabolites were identified (see Table 2). In the BPC plots of typical samples from each region, there were differences in metabolic composition between them in both positive ion (e.g., in Figure 2(c)) and negative ion (e.g., in Figure 2(d)) modes. In the PCA plot (e.g., in Figure 2(e)), the overall profile of the distribution of *D. officinale* samples from different regions could be observed.

### 3.3. Discovery of Differential Metabolites of *D. officinale* in Different Regions

**Figure 3:**
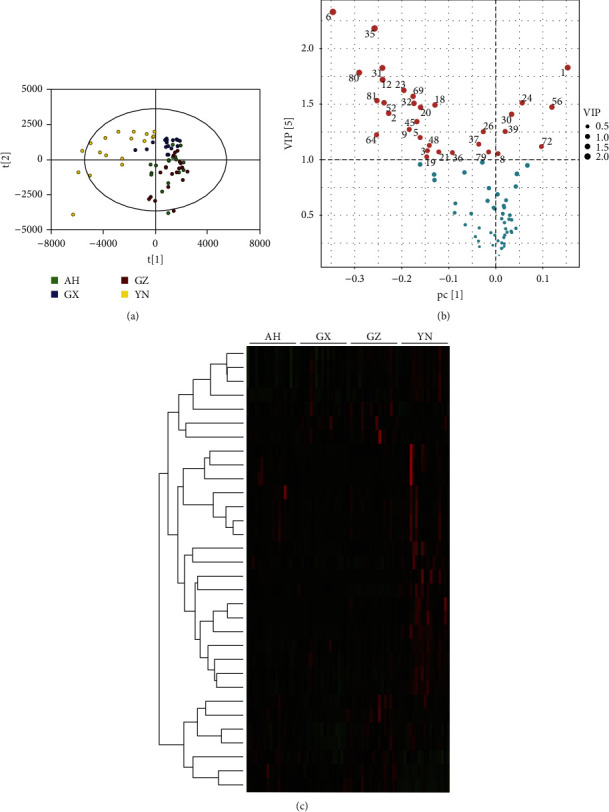
Discovery of differential components of *D. officinale* from different regions. (a) PLS-DA of *D. officinale* from four regions. (b) VIP plot of *D. officinale* from four regions. (c) Heatmap of *D. officinale* from four regions.

To further reveal the differences in the chemical composition of *D. officinale* in different regions, multivariate statistical analysis was used for the analysis. The PLS-DA model developed had good predictive power (R2X (cum) = 0.698, R2Y (cum) = 0.668) and confidence (Q2 (cum) = 0.504) (e.g., in Figure 3(a)). The results showed that YN and GX clustered separately by region, while the metabolite profiles of the two regions AH and GZ crossed obviously, indicating that the metabolites of *D. officinale* in the two regions have a similar expression. 32 differential metabolites were screened according to the variable importance for the projection (VIP) value > 1 (e.g., in Figure 3(b)). Further cluster heatmap analysis was done for these differential metabolites (e.g., in Figure 3(c)), and overall, the region with more differential metabolites was YN, followed by GZ, AH, and GX in that order.

### 3.4. Identification of Differential Compositions in D*. officinale* from Different Regions

**Figure 4:**
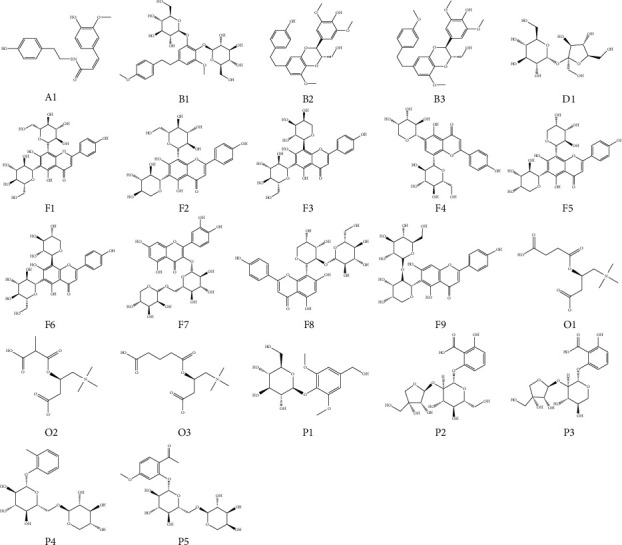
Chemical structures of differential compositions in *D. officinale* from four regions.

Based on the analysis of the extracted ion chromatograms and the results of VIP >1, 22 compounds with clear chemical structures were tentative identified (see Figure 4), including 1 amides (cis-N-feruloyltyramine: A1), 3 bibenzyls (dendromoniliside E; B1, dendrocandin U: B2, dendrocandin B: B3), 1 disaccharide (sucrose: D1), 9 flavonoids (vicenin 2: F1, vicenin 1: F2, schaftoside: F3, isoschaftoside: F4, apigenin 6,8-di-C-arabinoside: F5, vicenin 3: F6, rutin: F7, apigenin 8-C-*α*-L-arabinopyranosyl-2”-O-*β*-D-glucopyranoside: F8, apigenin 6-C-*α*-L-arabinopyranosyl-2”-O-*β*-D-glucopyranoside: F9), 3 organic nitrogenous compounds (succinylcarnitine: O1, O-methylmalonyl-L-carnitine: O2, O-glutaroyl-L-carnitine: O3), 5 phenolic glycosides (di-O-methylcrenatin: P1, 2,6-dihydroxybenzoic acid 2-O-*β*-D-apiofuranosyl-(1⟶2)-*β*-D-glucopyranoside: P2, 2,6-dihydroxybenzoic acid 2-O-*β*-D-apiofuranosyl-(1⟶2)-*β*-D-xylopyranoside: P3, 2-methylphenyl-O-*β*-D-xylopyranosyl-(1⟶6)-O-*β*-D-glucopyranoside: P4, paeonolide: P5).

**Figure 5:**
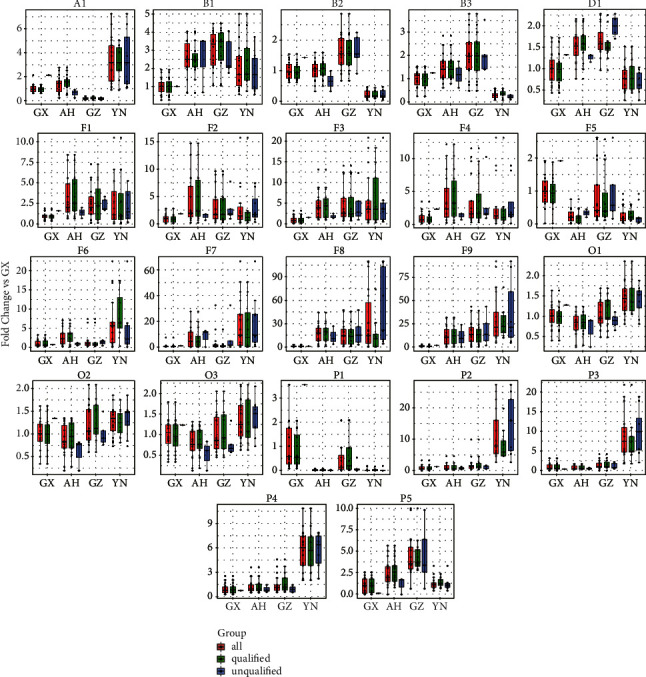
Expression of differential compositions in *D. officinale* from four regions.

In terms of the distribution of expression of these differential compositions (see Figure 5), the highest expression of differential compositions was still YN, followed by GZ, AH, and GX in that order. Among flavonoids, the higher expression regions were YN and AH. It is also evident that this differential composition was almost absent in GX samples. Among the phenolic glycosides, the higher expression regions were YN and GX. In the organic nitrogenous compounds and amides, the highest expression region was YN. In the bibenzyls and disaccharide, the higher expression regions was GZ.

## 4. Discussion

*D. officinale*, as a widely used valuable Chinese herbal medicine, adopts polysaccharides as its main active ingredient and quality control standard in the Ch.P. In the present study, we selected *D. officinale* from different origins of greenhouse cultivation with relatively stable quality as the research object, and firstly analyzed their polysaccharide content according to the Ch.P. method. The results showed that in terms of polysaccharide content, the order was GX > AH > GZ > YN. It has been observed that the production of polysaccharides in wolfberry and lingonberries decreases gradually, with the increase in altitude and decrease in temperature [19,20]. This has similarity with our results, in that GX (average altitude 97 m) and AH (average altitude 80 m) were at lower altitude and they both had high polysaccharide content, followed by GZ (average altitude 895 m), YN (average altitude 1116 m), respectively.

Previous literature reported that *D. officinale* is rich in flavonoids, phenanthrenes, bibenzyl, and other small molecule chemical components in addition to polysaccharides [21–24]. Therefore, it is unsystematic and incomplete to use polysaccharide content alone as a criterion for the medicinal value and quality evaluation of *D. officinale*. Herein, we used metabolomic analysis, combined with heat map and hierarchical clustering analysis, to reveal the differences in small molecule chemical composition among *D. officinale* from four different origins. The results elucidated that 22 compounds, including 1 amides (Cis-N-Feruloyltyramine), 3 bibenzyls (Dendromoniliside E, Dendrocandin U, Dendrocandin B), 1 disaccharide (Sucrose), 9 flavonoids (Vicenin 1, Vicenin 2, Vicenin 3, Schaftoside, Isoschaftoside, Rutin, Apigenin 6,8-di-C-arabinoside, Apigenin 6-C-*α*-L-arabinopyranosyl-2”-O-*β*-D-glucopyranoside, apigenin 8-C-*α*-L-arabinopyranosyl-2”-O-*β*-D-glucopyranoside), 3 organic nitrogenous compounds (Succinylcarnitine, O-Methylmalonyl-L-carnitine, O-Glutaroyl-L-carnitine), 5 phenolic glycosides (Di-O-methylcrenatin, Paeonolide, 2,6-Dihydroxybenzoic acid 2-O-*β*-D-apiofuranosyl-(1⟶2)-*β*-D-glucopyranoside, 2,6-Dihydroxybenzoic acid 2-O-*β*-D-apiofuranosyl-(1⟶2)-*β*-D-xylopyranoside, 2-Methylphenyl-O-*β*-D-xylopyranosyl-(1⟶6)-O-*β*-D-glucopyranoside) were the main differential chemical constituents in *D. officinale* from different origins. The growth environment of *D. officinale* cultivated in greenhouses is relatively controlled, and thus the differences in the chemical composition of *D. officinale* from these different regions are more from the influence of origin.

Among the flavonoids, most of them were flavone-C-glycosides. In plants, flavonoids have a variety of biological functions, such as regulating cell growth, enhancing nutrient recycling, and resisting biotic and abiotic stresses [25,26]. A flavonoids-rich diet not only helps prevent some chronic diseases, but it also has biological activities such as anti-inflammatory, anticancer, cardiovascular protection, and blood lipid regulation [27–29]. It has been reported that *Astragalus* polysaccharides significantly improve the solubility, stability, and solubilizing effect of flavonoids [30]. Synergistic effects of polysaccharides with flavonoids have been shown, which may contribute to better pharmacological effects. However, almost all the differential flavonoids were higher in the YN sample with the lowest polysaccharide content, while the opposite result was observed in the GX sample with the highest polysaccharide content. And, it was corroborated in the YN samples from the samples with qualified and unqualified polysaccharide contents. This also reinforces that the evaluation of the quality of *D. officinale* by polysaccharide content alone is imperfect, and the content of small molecule components needs to be examined systematically.

Bibenzyls and disaccharide were mainly highly expressed in GZ. The bibenzyls is one of the active ingredients in *Dendrobium* genus. The main pharmacological activities identified in the compounds are anti-tumor, antidiabetic, antiplatelet aggregation, anti-inflammatory, etc [31–33]. It is expressed that the bibenzyls had a wide range of medicinal effects in *Dendrobium* genus. Sucrose, a disaccharide, is the main product of photosynthesis and can act as a signaling molecule for a wide range of plant growth processes [34]. It has specific functions in plant metabolism, growth, and development. Phenolic glycosides, amides, and organic nitrogenous compounds were the main sign component of YN. Of these, phenolic glycosides are widely distributed in plants and are phenylalanine and tyrosine metabolites with antimalarial, antineuroinflammatory, antiobesity and antioxidant, and other activities, mainly [35–38]. It has lower biological activity compared to the corresponding glycosides, some of which may later be used as nutritional agents or adjuvants [35]. Some studies have shown that the accumulation of total phenols may be positively correlated with altitude gradient [39]. It has been observed that succinylcarnitine (O1) correlates with total cholesterol or LDL, activated partial thromboplastin time [40]. These studies also predicted that *D. officinale* containing different components is suitable for different disease treatment.

## 5. Conclusion

In summary, this study identified the differential compositions of *D. officinale* cultivated in greenhouses in four regions of China (AH, GX, GZ, YN). The results showed that the polysaccharide content in *D. officinale* was strongly related to its growing altitude, with the highest average polysaccharide content in the GX sample (altitude: 97 m) and the lowest average polysaccharide content in the YN sample (altitude: 1116 m). There was also a significant difference in the small molecule chemical composition in *D. officinale*, where the content of flavonoids showed an opposite trend to that of polysaccharides. These results indicated that the current quality standard for evaluating the quality of *D. officinale* by polysaccharide content alone is imperfect, and small molecule compounds need to be included as quality markers.

## Figures and Tables

**Table 1:** Information of *D. officinale* samples.

No.	Collecting samples	Sample numbers	Average altitude (*m*)	Longitude (*E*)	Latitude (*N*)
1	AH	18	80	116°19′33″	31°23′20″
2	GX	17	97	110°38′20″	22°49′48″
3	GZ	18	895	107°65′76″	26°21′16″
4	YN	18	1116	99°42′55″	22°24′10″

**Table 2:** Secondary metabolites of *D. officinale* from four regions.

No.	Identification	RT (min)	Add ion	m/z	ppm	Molecular formula
1	Sucrose	0.587	[M-H]^−^	341.1092	0.7	C_12_H_22_O_11_
2	Succinylcarnitine	0.628	[M+H]^+^	262.1292	2.6	C_11_H_19_NO_6_
3	O-methylmalonyl-L-carnitine	0.755	[M+H]^+^	262.1291	2.4	C_11_H_19_NO_6_
4	Guanosine	0.807	[M-H]^−^	282.0838	−2.0	C_10_H_13_N_5_O_5_
5	O-glutaroyl-L-carnitine	0.898	[M+H]^+^	276.1453	4.0	C_12_H_21_NO_6_
6	2-Methyl-1H-indol-7-yl-*β*-D-mannopyranoside or isomer	1.416	[M+H]^+^	310.1289	1.4	C_15_H_19_NO_6_
7	Protocatechuic acid-3-O-glucoside	1.452	[M-H]^−^	315.0716	−1.7	C_13_H_16_O_9_
8	di-O-methylcrenatin	1.741	[M+H]^+^	347.1313	−6.9	C_15_H_22_O_9_
9	2,6-Dihydroxybenzoic acid 2-O-*β*-D-apiofuranosyl-(1⟶2)-*β*-D-glucopyranoside	1.915	[M-H]^−^	447.1136	−1.8	C_18_H_24_O_13_
10	Benzyl-*β*-gentiobioside	1.992	[M-H]^−^	431.1549	−2.3	C_19_H_28_O_11_
11	Dendromoniliside C	2.251	[M-H]^−^	443.1912	−2.4	C_21_H_32_O_10_
12	2,6-Dihydroxybenzoic acid 2-O-*β*-D-apiofuranosyl-(1⟶2)-*β*-D-xylopyranoside	2.306	[M-H]^−^	417.1031	−1.8	C_17_H_22_O_12_
13	Syringin	2.408	[M+Na]^+^	395.1310	−0.5	C_17_H_24_O_9_
14	Dihydrosyringin	2.480	[M+Na]^+^	397.1470	0.3	C_17_H_26_O_9_
15	Khaephuoside A	2.526	[M+Na]^+^	501.1575	−0.7	C_20_H_30_O_13_
16	Gastrodin	2.612	[M+Na]^+^	309.0946	0.3	C_13_H_18_O_7_
17	2-Methoxyphenyl-1-O-*β*-D-apiofuromosyl-(1⟶2)-*β*-D-glucopyranside	2.637	[M-H]^−^	417.1393	−2.4	C_18_H_26_O_11_
18	Vicenin 2	2.654	[M-H]^−^	593.1503	−1.5	C_27_H_30_O_15_
19	2-methylphenyl-O-*β*-D-xylopyranosyl-(1⟶6)-O-*β*-d-glucopyranoside	2.709	[M-H]^−^	401.1446	−1.7	C_18_H_26_O_10_
20	Vicenin 1	2.797	[M-H]^−^	563.1399	−1.3	C_26_H_28_O_14_
21	Schaftoside	2.858	[M-H]^−^	563.1398	−1.5	C_26_H_28_O_14_
22	Dihydromelilotoside	2.880	[M-H]^−^	327.1079	−1.9	C_15_H_20_O_8_
23	Isoschaftoside	2.951	[M-H]^−^	563.1398	−1.4	C_26_H_28_O_14_
24	Syringaresinol 4,4'-di-O-*β*-D-glucopyranoside	2.970	[M+Na]^+^	765.2566	−1.4	C_34_H_46_O_18_
25	Apigenin 6-C-*β*-D-xylopyranosyl-8-C-*α*-L-arabinopyranoside	3.012	[M-H]^−^	533.1292	−1.6	C_25_H_26_O_13_
26	Dendromoniliside E	3.026	[M+Na]^+^	621.2147	−1.1	C_28_H_38_O_14_
27	Shashenoside I	3.048	[M+Na]^+^	527.1734	−0.2	C_22_H_32_O_13_
28	Violanthin	3.084	[M-H]^−^	577.1555	−1.3	C_27_H_30_O_14_
29	Dendrobine	3.114	[M+H]^+^	264.1957	−0.4	C_16_H_25_NO_2_
30	Apigenin 6,8-di-C-arabinoside	3.106	[M-H]^−^	533.1291	−1.8	C_25_H_26_O_13_
31	Vicenin 3	3.111	[M-H]^−^	563.1394	−2.2	C_26_H_28_O_14_
32	Rutin	3.111	[M-H]^−^	609.1455	−1.1	C_27_H_30_O_16_
33	Apigenin-7-O-*β*-D-glucoside	3.161	[M-H]^−^	431.0972	−2.6	C_21_H_20_O_10_
34	Apigenin 6-C-*α*-L-arabinopyranosyl-8-C-*β*-D-xylopyranoside	3.238	[M-H]^−^	533.1291	−1.8	C_25_H_26_O_13_
35	Apigenin 8-C-*α*-L-arabinopyranosyl-2”-O-*β*-D-glucopyranoside	3.381	[M-H]^−^	563.1396	−1.8	C_26_H_28_O_14_
36	Apigenin 6-C-*α*-L-arabinopyranosyl-2”-O-*β*-D-glucopyranoside	3.425	[M-H]^−^	563.1399	−1.3	C_26_H_28_O_14_
37	Syringaresinol *β*-D-glucoside	3.522	[M+Na]^+^	605.2197	−1.2	C_28_H_38_O_13_
38	N-p-cis-coumaroyltyramine	3.742	[M+H]^+^	284.1282	0.3	C_17_H_17_NO_3_
39	Paeonolide	3.767	[M-H]^−^	459.1501	−1.5	C_20_H_28_O_12_
40	N-trans-feruloyltyramine	3.930	[M+H]^+^	314.1387	−0.1	C_18_H_19_NO_4_
41	Dendrocandin E	3.894	[M-H]^−^	275.0917	−2.8	C_15_H_16_O_5_
42	icariol A_2_-4-O-*β*-D-glucopyranoside	4.007	[M+Na]^+^	621.2141	−2.0	C_28_H_38_O_14_
43	4,4'-dihydroxy-3,5-dimethoxybibenzyl	4.010	[M-H]^−^	273.1124	−3.1	C_16_H_18_O_4_
44	N-p-trans-coumaroyltyramine	4.012	[M+H]^+^	284.1280	−0.2	C_17_H_17_NO_3_
45	cis-N-Feruloyltyramine	4.128	[M+H]^+^	314.1385	−0.5	C_18_H_19_NO_4_
46	Dendrocandin C	4.175	[M-H]^−^	289.1075	−2.1	C_16_H_18_O_5_
47	3',5,5',7-tetrahydroxyflavanone	4.214	[M-H]^−^	287.0554	−2.6	C_15_H_12_O_6_
48	Bornyl-2-O-*β*-D-arabinofuranosyl(1⟶6)-*β*-D-glucopyranoside	4.379	[M-H]^−^	447.2228	−1.7	C_21_H_36_O_10_
49	Tristin	4.440	[M-H]^−^	259.0971	−1.8	C_15_H_16_O_4_
50	Naringenin	4.699	[M-H]^−^	271.0608	−1.6	C_15_H_12_O_5_
51	trans-Cinnamoyl-p-hydroxybenzenethylamine	5.005	[M+H]^+^	268.1333	0.5	C_17_H_17_NO_2_
52	Dendrocandin O	5.032	[M+H]^+^	455.1696	−1.0	C_25_H_26_O_8_
53	Trihydroxyoctadecaenoic acid	5.052	[M-H]^−^	329.2329	−1.4	C_18_H_34_O_5_
54	Dendrocandin M	5.109	[M+Na]^+^	493.1832	−0.2	C_26_H_30_O_8_
55	Dendrocandin K	5.123	[M-H]^−^	515.1702	−1.9	C_30_H_28_O_8_
56	Dendrocandin U	5.231	[M+H]^+^	469.1856	−0.3	C_26_H_28_O_8_
57	Dendrocandin N	5.264	[M+H]^+^	439.1751	−0.1	C_25_H_26_O_7_
58	Dendrocandin T	5.286	[M+Na]^+^	521.1779	−0.7	C_27_H_30_O_9_
59	Syringaresinol	5.324	[M+Na]^+^	441.1518	−0.5	C_22_H_26_O_8_
60	Dendrocandin R	5.377	[M-H]^−^	329.1386	−2.7	C_19_H_22_O_5_
61	Dengraol A	5.377	[M-H]^−^	499.1749	−2.6	C_30_H_28_O_7_
62	Dendrocandin H	5.377	[M-H]^−^	525.1179	−2.3	C_30_H_22_O_9_
63	3,4-Dihydroxy-5,4'-dimethoxybibenzyl	5.415	[M-H]^−^	273.1127	−2.0	C_16_H_18_O_4_
64	Erianin	5.434	[M+H]^+^	319.1540	0.0	C_18_H_22_O_5_
65	15-Methylhexadecaphytosphingosine or isomer	5.473	[M+H]^+^	304.2861	4.8	C_17_H_37_NO_3_
66	Dendrocandin G	5.476	[M-H]^−^	529.1856	−2.4	C_31_H_30_O_8_
67	Dendrocandin L	5.548	[M-H]^−^	509.1226	−3.2	C_30_H_22_O_8_
68	Physcion	5.567	[M+H]^+^	285.0759	0.4	C_16_H_12_O_5_
69	Dendrocandin J	5.548	[M-H]^−^	529.1854	−2.6	C_31_H_30_O_8_
70	2-Amino-octadecane-1,3,4-triol	5.589	[M+H]^+^	318.3010	2.4	C_18_H_39_NO_3_
71	Dendrocandin I	5.680	[M-H]^−^	543.1999	−4.6	C_32_H_32_O_8_
72	3-Methoxy-5-[2-(4-methoxyphenyl) ethyl] phenol	5.666	[M+H]^+^	259.1331	0.8	C_16_H_18_O_3_
73	Dendrocandin B	5.677	[M+H]^+^	483.2014	0.1	C_27_H_30_O_8_
74	2,3,4,7-Tetramethoxyphenanthrene	5.705	[M+H]^+^	299.1278	0.0	C_18_H_18_O_4_
75	Sphinganine	5.727	[M+H]^+^	302.3057	1.0	C_18_H_39_NO_2_
76	Dendrocandin F	5.823	[M-H]^−^	543.2010	−2.7	C_32_H_32_O_8_
77	Linolenic acid	5.986	[M+H]^+^	279.2321	0.8	C_18_H_30_O_2_
78	Linoleic acid	6.104	[M-H]^−^	279.2320	−3.4	C_18_H_32_O_2_
79	Aphanamixoid J or isomer	6.531	[M+H]^+^	609.2704	1.6	C_34_H_40_O_10_
80	Aphanamixoid J or isomer	6.614	[M+H]^+^	609.2722	4.5	C_34_H_40_O_10_
81	Aphanamixoid G or isomer	6.702	[M+H]^+^	593.2765	3.4	C_34_H_40_O_9_
82	Aphanamixoid G or isomer	6.818	[M+H]^+^	593.2747	0.4	C_34_H_40_O_9_

*Note.* A: amides; B: bibenzyls; D: disaccharide; F: flavonoids; O: organic nitrogenous compounds; P: phenolic glycosides.

## Data Availability

The original contributions presented in the study are included in the article/Supplementary Material, further inquiries can be directed to the corresponding authors.

## Supplementary Materials

Supplementary Materials The description of the supplementary materials is as follows: This is the detailed information of secondary metabolites of *D. officinale* from four regions, supplemented with molecular weight and MS/MS information.
